# Supplementary material for: Clostridium butyricum Potentially Improves Immunity and Nutrition through Alteration of the Microbiota and Metabolism of Elderly People with Malnutrition in Long-Term Care
Source: Nutrients. 2022 Aug 28;14(17):3546. doi: 10.3390/nu14173546 (PMC9460359; doi:10.3390/nu14173546)

Supplementary figures for:

Clostridium butyricum Potentially Improves Immunity and Nutrition through Alteration of the Microbiota and Metabolism of Elderly People with Malnutrition in Long-Term Care

Supplementary figure S1. Differential gut microbiota species enriched in Test Group. **a.** The differential species enriched in T3 identified by wilcoxon rank-sum test (P<0.05) of T0 and T3 groups; **b.** The differential species enriched in T3 identified by Kruskal-Wallis test (P<0.05) among T0, T3 and T6 groups.

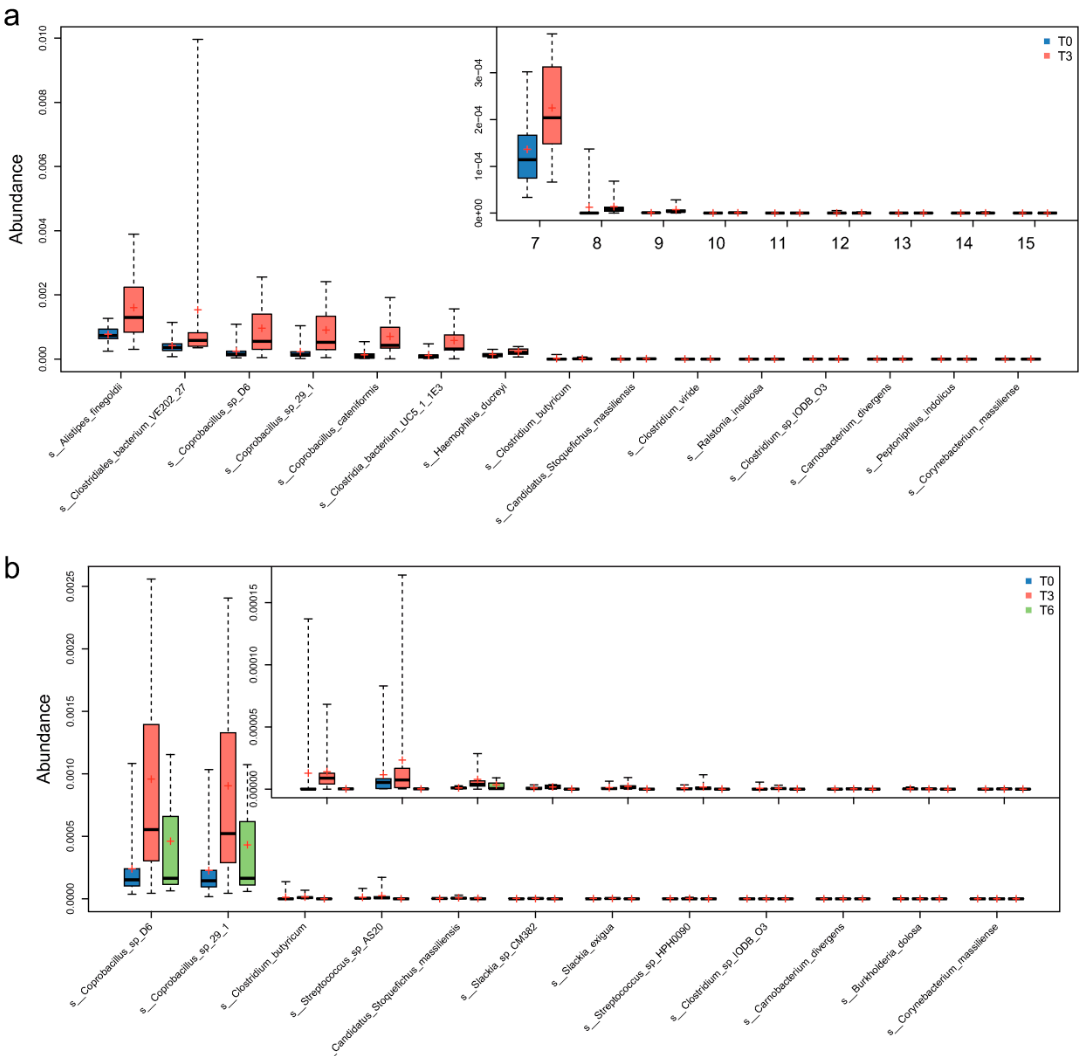

Supplementary figure S2. Differential gut microbiota species enriched in Control Group, for base line studies along seasonal change. **a.** The species enriched in C3 were identified by wilcoxon rank-sum test (P<0.05) between C0 and C3 groups; **b.** The differential species enriched in T3 identified by Kruskal-Wallis test (P<0.05) among C0, C3 and C6 groups; The intersection of the differential species in **a** and **b** were the the base line of fluctuations along seasonal change (**c**).

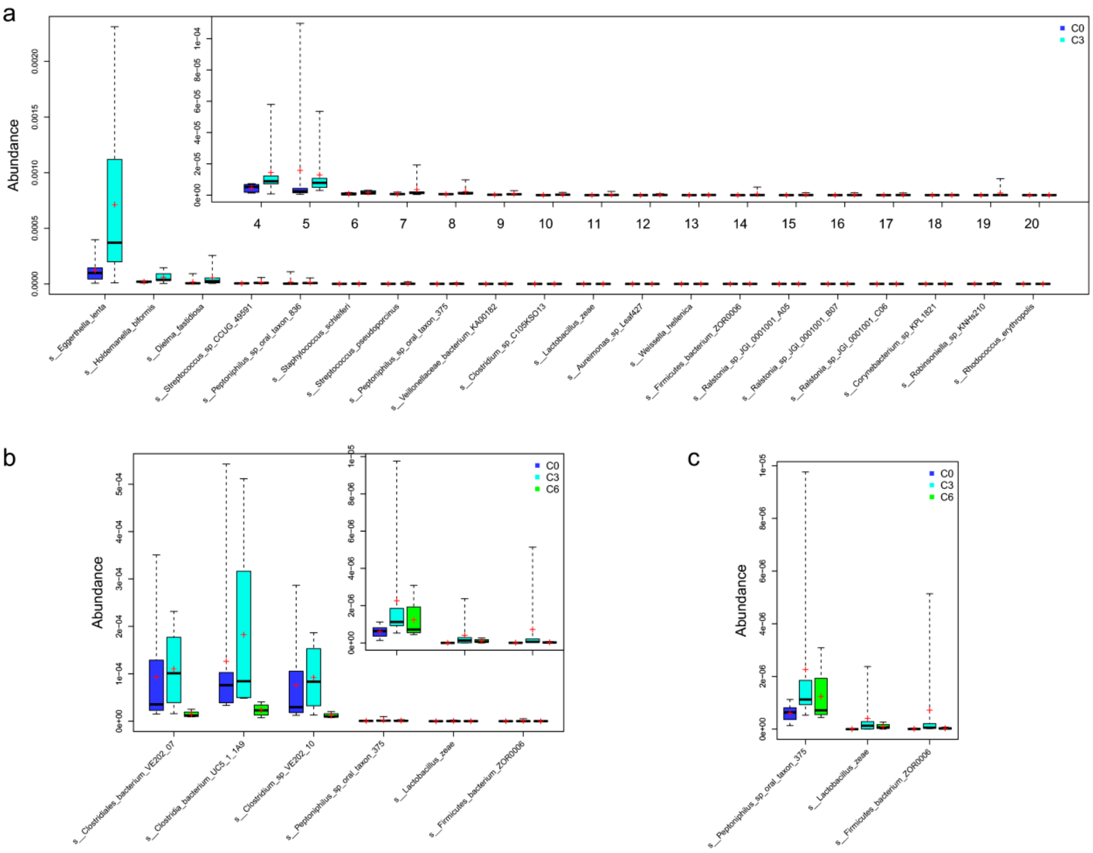

Supplementary figure S3. Box-plot of abundant beneficial intestinal bacteria. The boxes represent the interquartile range (IQR), from the first and third quartiles, and the inside line represents the median. The whiskers denote the lowest and highest values within 1.5 IQR from the first and third quartiles. The circles represent outliers beyond the whiskers.

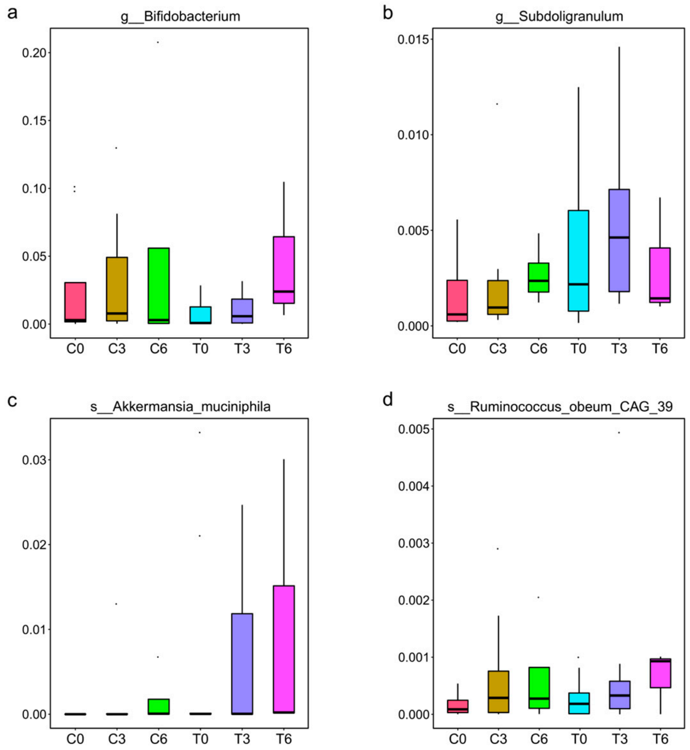

Supplementary figure S4. The differential KOs enriched in T0 and C0 groups. a. The differential KOs enriched in T0 were identified by wilcoxon rank-sum test (P<0.05) between T0 and T3 groups; b. The differential KOs enriched in C0 were identified by wilcoxon rank-sum test (P<0.05) between C0 and C3 groups. c. The KEGG classification of the differential KOs enriched in T0 group with the Level 1 and Level 2 information; d. The KEGG classification of the differential KOs enriched in C0 group with the Level 1 and Level 2 information.

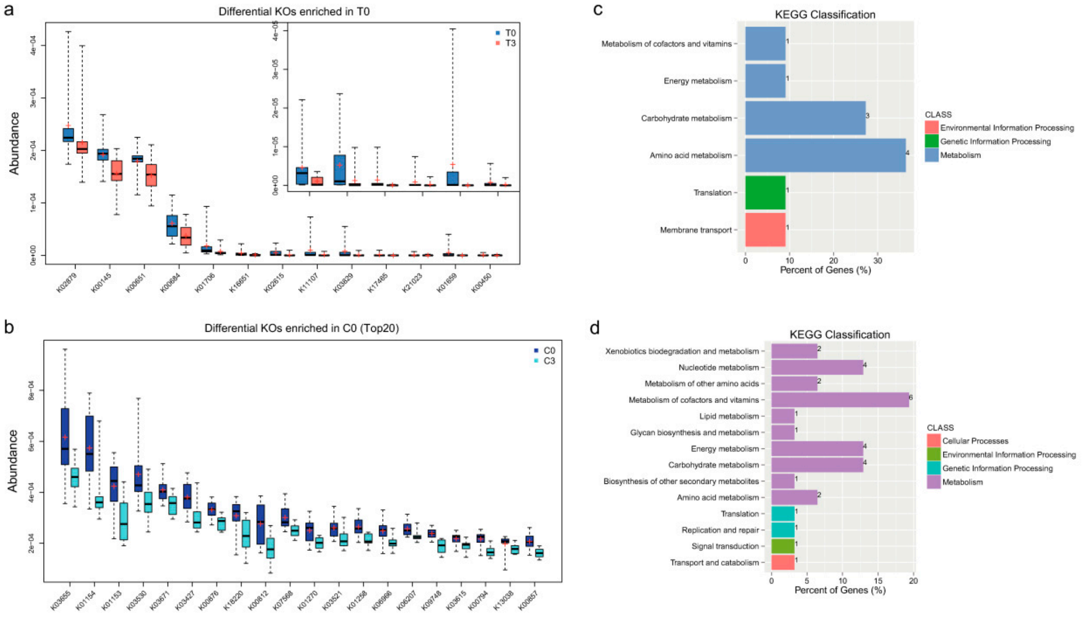

Supplementary figure S5. Correlation analysis between the differential species (horizontal axis) and the differential KOs (longitudinal axis) by spearman correlation coefficient. Color indicates the strength of correlation, the red indicates the positive correlation, and the blue indicates the negative correlation. An asterisk '+' means *P*-value<0.05, and an asterisk '*' means *P*-value<0.01. Red text indicates differential KOs enriched in T3, blue text indicates differential KOs enriched in T0.

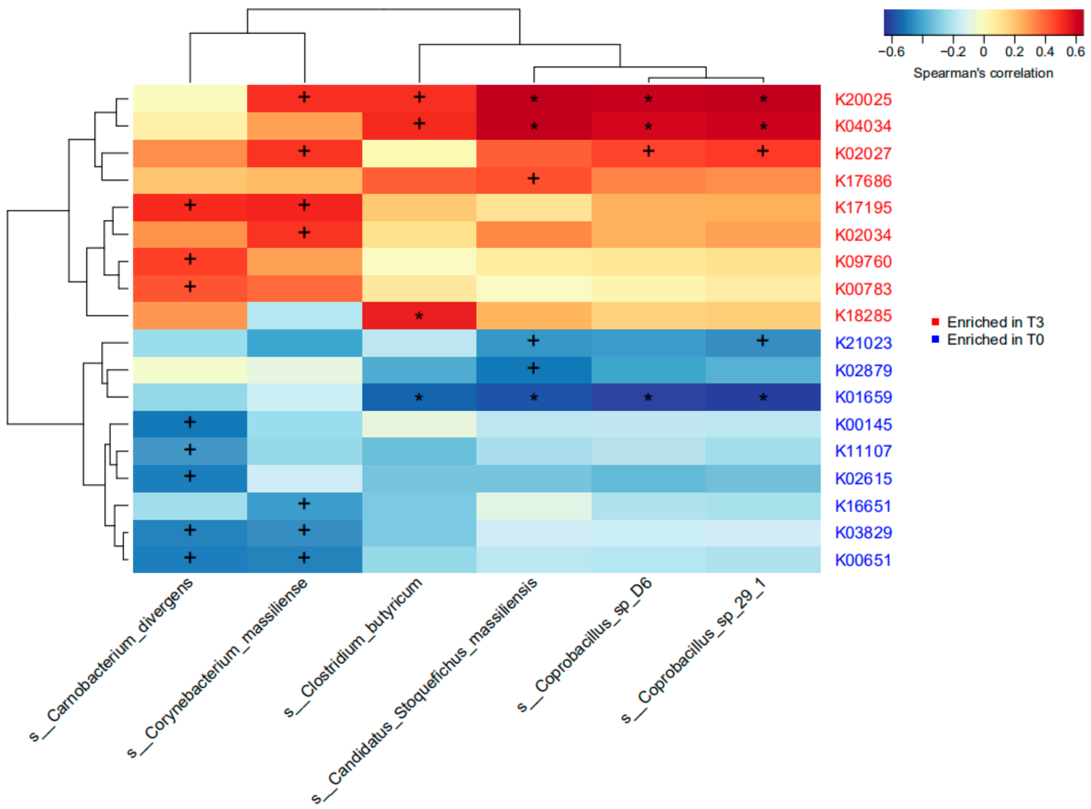

Supplementary figure S6. Correlation analysis between the differential KOs (longitudinal axis) and the (horizontal axis) (differential metabolites in 6a and all detected metabolites in 6b) by spearman correlation coefficient. Color indicates the strength of correlation, the red indicates the positive correlation, and the blue indicates the negative correlation. An asterisk '+' means *P*-value<0.05, and an asterisk '*' means *P*-value<0.01. Red text indicates differential KOs enriched in T3, blue text indicates differential KOs enriched in T0.

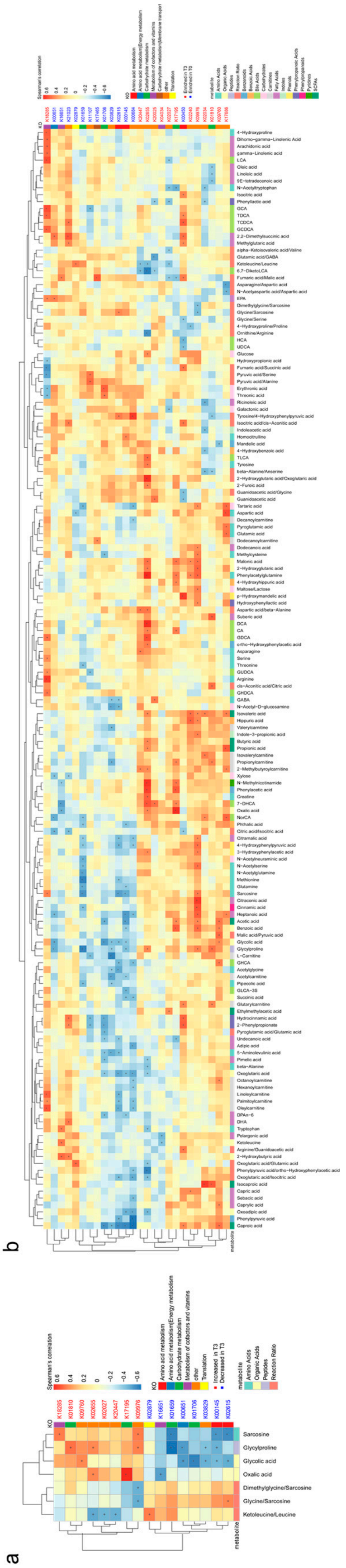

Supplementary figure S7. Box-plot of five clinical biomarkers indicative of gut integrity. The boxes represent the interquartile range (IQR), from the first and third quartiles, and the inside line represents the median. The whiskers denote the lowest and highest values within 1.5 IQR from the first and third quartiles. The circles represent outliers beyond the whiskers. Wilcoxon rank-sum test was used.

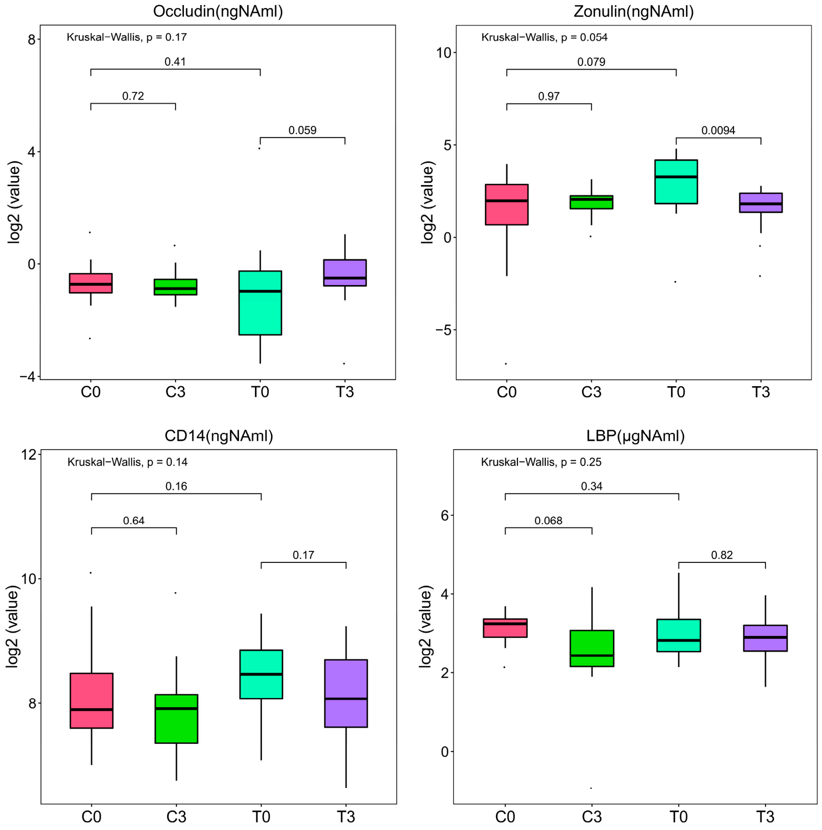

Supplementary figure S8. The Principal Co-ordinates Analysis (PCoA) plot showed the comparison of the microbiota structure between subjects of this study and the latest available metagenome databases from 7 cohorts. The 7 cohorts were IBD patients, RA patients, metabolic syndrome patients, fatty liver patients, T2D patients, longevous participants and their offsprings.

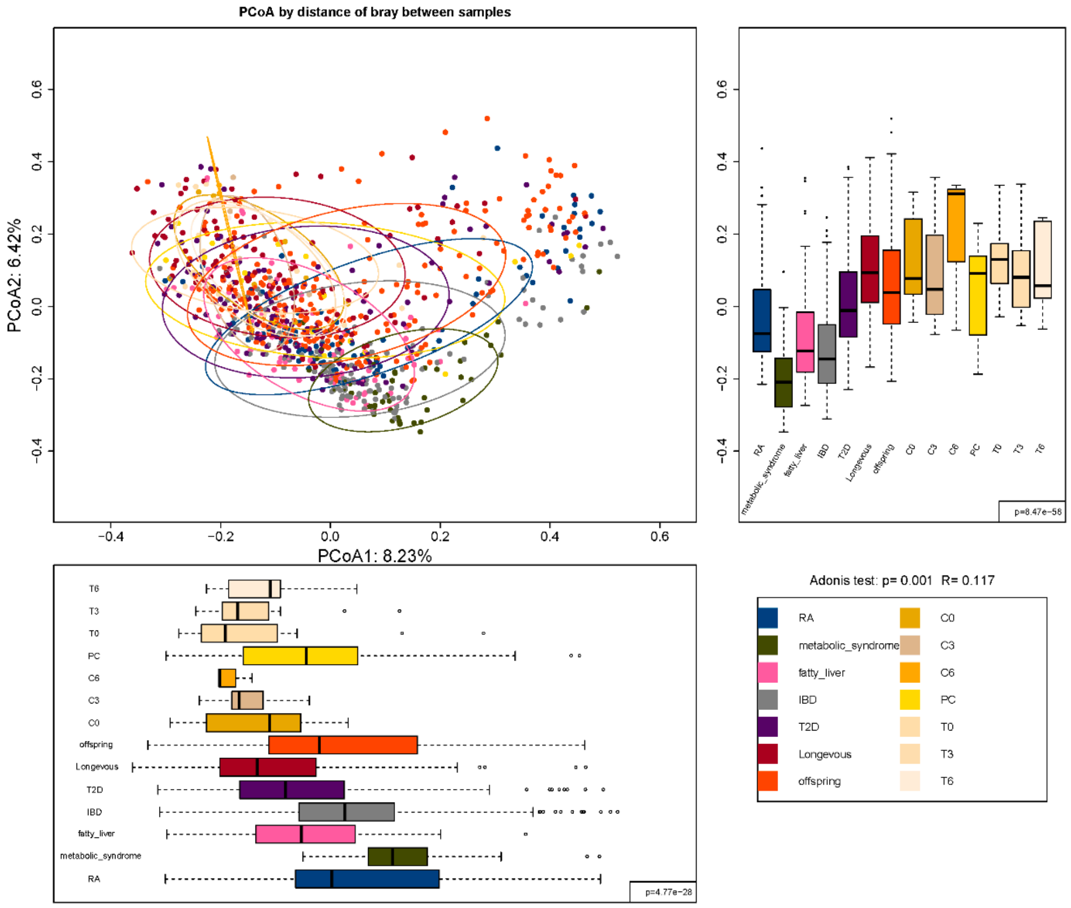

Supplementary figure S9. The association analysis between metabolites and clinical phenotypes using MaAsLin (FDR < 0.1). This analysis indicates that the human metabolism is in part associated with age and gender but not disease or BMI.

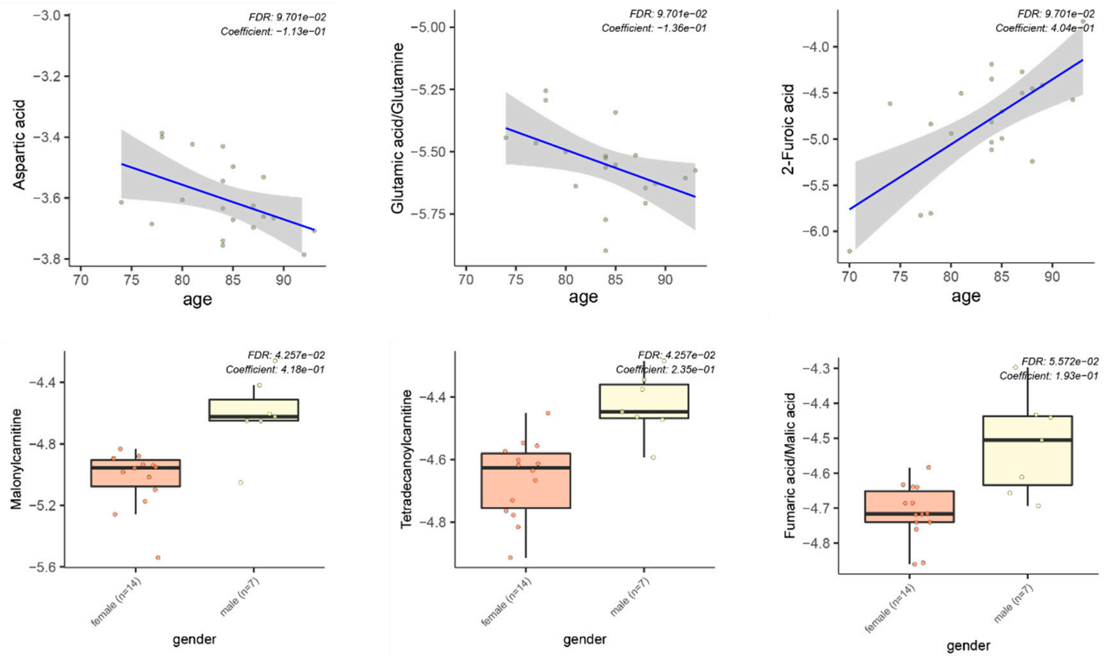

Supplement: Supplementary file 1 [file nutrients-14-03546-s001.zip › final Supplementary figures.pdf]
